# D-MEKK1, the *Drosophila *orthologue of mammalian MEKK4/MTK1, and *Hemipterous*/D-MKK7 mediate the activation of D-JNK by cadmium and arsenite in Schneider cells

**DOI:** 10.1186/1471-2121-7-7

**Published:** 2006-02-01

**Authors:** Olga P Ryabinina, Ezhilkani Subbian, Mihail S Iordanov

**Affiliations:** 1 Department of Cell and Developmental Biology, Oregon Health & Science University, Oregon 97239, Portland; 2 Department of Biochemistry and Molecular Biology, Oregon Health & Science University, Oregon 97239, Portland

## Abstract

**Background:**

The family of c-Jun NH_2_-terminal kinases (JNK) plays important roles in embryonic development and in cellular responses to stress. Toxic metals and their compounds are potent activators of JNK in mammalian cells. The mechanism of mammalian JNK activation by cadmium and sodium arsenite involves toxicant-induced oxidative stress. The study of mammalian signaling pathways to JNK is complicated by the significant degree of redundancy among upstream JNK regulators, especially at the level of JNK kinase kinases (JNKKK).

**Results:**

Using *Drosophila melanogaster *S2 cells, we demonstrate here that cadmium and arsenite activate *Drosophila *JNK (D-JNK) via oxidative stress as well, thus providing a simpler model system to study JNK signaling. To elucidate the signaling pathways that lead to activation of D-JNK in response to cadmium or arsenite, we employed RNA interference (RNAi) to knock down thirteen upstream regulators of D-JNK, either singly or in combinations of up to seven at a time.

**Conclusion:**

D-MEKK1, the fly orthologue of mammalian MEKK4/MTK1, and *Hemipterous*/D-MKK7 mediates the activation of D-JNK by cadmium and arsenite.

## Background

Mitogen-activated protein (MAP) kinases are involved in fundamental biological processes, such as cell proliferation, differentiation, migration, and death, as well as in various aspects of embryonic development, morphogenesis, inflammation, wound healing, and cellular responses to stress [1-11]. The mammalian MAPK superfamily encompasses the extracellular signal-regulated kinases (ERK1 and ERK2), the p38 MAP kinases (p38α, β, γ, and δ), the big MAP kinase (BMK1/ERK5), and the c-Jun NH_2_-terminal kinases (JNK1, JNK2, and JNK3) [1-7]. The JNK family of kinases is of particular importance for cellular responses to stress [8-11]. Most somatic cells express JNK1 and JNK2, the expression of JNK3 being restricted predominantly to the brain [12,13]. While the single homozygous deletions of either *jnk1 *or *jnk2 *in the mouse revealed a significant functional redundancy between JNK1 and JNK2, the compound *jnk1*^-/-^/*jnk2*^-/- ^mutant mice die before birth, thus revealing the non-redundant role of the JNK family as a whole in development [14]. In addition to development, JNKs play important roles in mediating cellular responses to oxidative, genotoxic, ribotoxic, and hyperosmotic stresses [3,15,16]. JNKs are activated via phosphorylation by two upstream JNK kinases (JNKK), MKK4 and MKK7 [8,17]. In turn, JNKK are activated via phosphorylation by JNK kinase kinases (JNKKK). To date, at least 13 mammalian JNKKK have been identified, namely: (*i*) the MEK kinase (MEKK) family, containing 4 members, MEKK1, 2, 3, and 4 [18], (*ii*) the apoptosis signal-regulating kinase 1 (ASK1) [19], (*iii*) the transforming growth factor β-activated kinase 1 (TAK1) [20], and (*iv*) the family of mixed lineage kinases (MLK), containing three subfamilies, namely the MLK subfamily (MLK 1, 2, 3, and 4), the dual-leucine-zipper-bearing kinase subfamily (DLK and LZK), and the zipper sterile-α-motif kinase (ZAK) [21]. Clearly, the complexity and specificity of JNK activation is executed mainly at the level of JNKKK, as the number of different JNKKK exceeds by far the number of JNKK. Upstream of JNKKK, the regulation of the JNK pathways is thought to be dependent on the activity of various small GTP-binding proteins (such as Rho, Rac, cdc42, Ras, and Ral) [22-25]. Thus, elucidation of the mechanisms of activation of JNK by a stressor of interest necessarily involves the identification of GTPase(s) and JNKKK relaying a stress-generated signal to JNKK and JNK.

Toxic metals and their compounds (e.g. cadmium and arsenite) are among the most potent activators of JNK [26-28]. Antioxidants prevent or reduce the activation of JNK by both cadmium and arsenite, suggesting that toxicant-induced oxidative stress is operative in the activation of JNK by these agents [28]. However, the identification of specific mammalian JNKKK-JNKK modules involved in the activation of JNK by cadmium and arsenite has been difficult due to the complexity and potential redundancy of mammalian JNKKK.

The genome of the fruit fly *Drosophila melanogaster *possesses all JNKKK, JNKK, and JNK families present in the mammalian genome, but represented, typically, by fewer genes. For instance, the mammalian MEKK family is represented in *Drosophila *by a single member, *mekk1*/D-MEKK1 [29]. The mammalian MLK family is represented in *Drosophila *by 2 members, *slpr*/D-MLK and *CG8789*/D-DLK [29]. The fruit fly orthologues of mammalian ASK1 and TAK1 are *pk92B*/D-ASK and *tak1*/D-TAK1, respectively [29]. The fruit fly orthologues of MKK4 and MKK7 are *mkk4*/D-MKK4 and *hep*/D-MKK7, respectively [29]. Finally, Drosophila contains only one JNK gene, *bsk*/D-JNK [29]. This evolutionary conservation of JNK signal transduction pathways among metazoans underscores the fundamental importance of JNK in mediating inducible stress responses.

With the above rationale in mind, we employed *Drosophila *S2 cells and the RNA interference (RNAi) technique (also known as "knock down" technique) for gene silencing [30,31] to investigate the signal transduction pathways mediating the activation of D-JNK by cadmium and arsenite. We knocked down 13 upstream regulators of D-JNK, either singly or in combinations of up to 7 at a time. As a result of this approach, we demonstrate the involvement of D-MEKK1 and D-MKK7 in the activation of D-JNK by cadmium and arsenite.

## Results

### Cadmium and arsenite activate D-JNK, D-p38 MAPK, and D-ERK in Drosophila S2 cells

**Figure 1:**
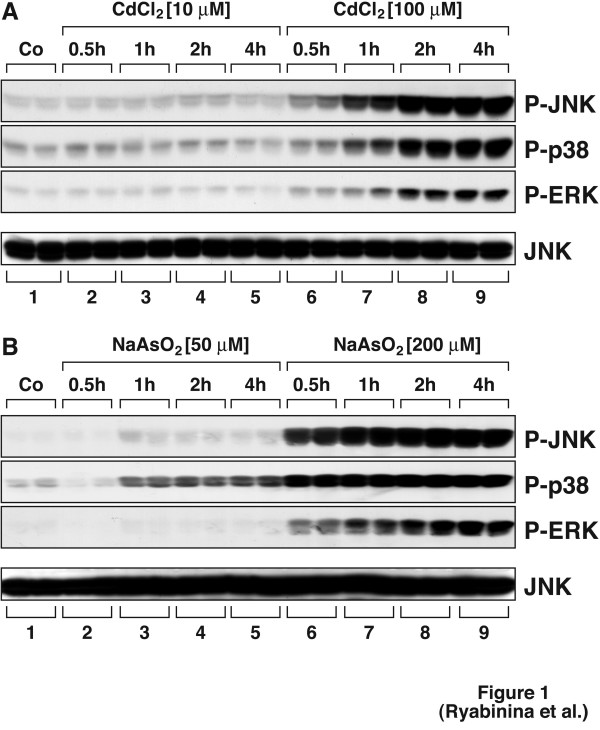
Cadmium and arsenite activate JNK, p38 MAPK, and ERK in S2 cells. Four million cells were plated in 1 ml DSFM per well on 6-well plates 2 days before treatments. Cells were left untreated (Co) or received 10 μM or 100 μM CdCl_2 _(A), or 50 μM or 200 μM NaAsO_2 _(B). Treatments were done in duplicates. Cells were harvested at the indicated times after the treatments and analyzed for MAP kinase phosphorylation in immunoblot analyses using phosphoepitope-specific antibodies as indicated. Immunoblotblot analysis using an antibody recognizing JNK indicates equal protein loading. An experiment representative of 3 repetitions is shown.

**Figure 2:**
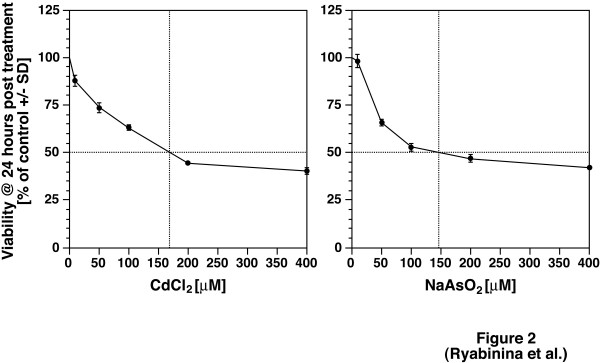
Dose-dependent toxicity of cadmium and arsenite in S2 cells. Four million cells were plated in 1 ml DSFM per well on 6-well plates 2 days before treatments. Cells were left untreated or received the indicated doses of either CdCl_2 _(left panel), or NaAsO_2 _(right panel). Twenty four hours later, 100 μl of cell suspension were used for viability assays using the CellTiter-Blue™ Reagent. Viability is presented as percentage of untreated cells (+/- SD). An experiment representative of 3 repetitions is shown.

**Figure 3:**
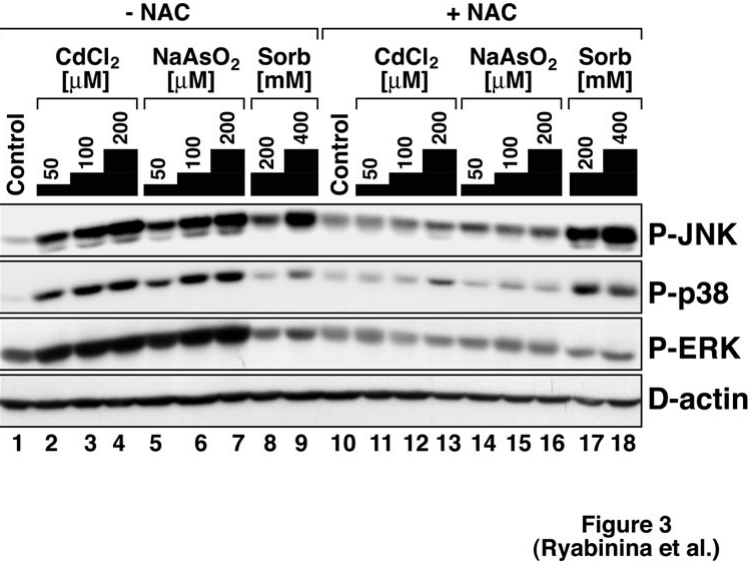
Cadmium and arsenite activate JNK, p38 MAPK, and ERK via toxicant-induced oxidative stress. Cells were plated as in Fig. 1. Thirty minutes before the treatments, 1 ml of DSFM or 1 ml of 60 mM buffered NAC in DSFM (pH 7.3; final concentration 30 mM) were added per well as indicated. Cells were harvested for MAP kinase phosphorylation analyses 2 hours after the indicated concentrations of CdCl_2 _or NaAsO_2 _and 30 min after the indicated concentrations of sorbitol. Control (without NAC-pretreatment, lane 1, or with NAC-pretreatment, lane 10) cells were harvested at the same time as the CdCl_2_- and NaAsO_2_-treated cells. D-actin was detected as loading control. An experiment representative of 3 repetitions is shown.

To elucidate the responses of *Drosophila *MAP kinases to toxic metals, we employed S2 cells, an immortalized cell line of larval hemolymph origin. Activation of MAP kinases was assessed in immunoblot assays using antibodies specific for the phosphorylated forms of D-JNK, D-p38 MAPK, and D-ERK. As shown in Fig. 1, both cadmium and arsenite triggered the phosphorylation of D-JNK, D-p38 MAPK, and D-ERK in a dose- and time-dependent manner. As significant activation of the three MAP kinases by either agent was achieved at relatively high doses (100 μM for cadmium and 200 μM for arsenite), we measured the overall toxicity to S2 cells of these doses of cadmium and arsenite. Figure 2 shows that 100 μM of cadmium triggered less than 40% cell death within 24 hours and that 200 μM of arsenite triggered ~50–55% cell death within 24 hours. Further experiments (Fig. 3) demonstrated that both cadmium and arsenite caused detectable phosphorylation of all three MAP kinases at doses as low as 50 μM. We concluded, therefore, that *Drosophila *MAP kinases respond to concentrations of cadmium and arsenite within and below the LD_50 _range of these agents.

### Cadmium and arsenite activate Drosophila MAP kinases via oxidative stress

To investigate whether reactive oxygen species (ROS) mediate the activation of *Drosophila *MAP kinases by cadmium and arsenite, we employed pretreatment of S2 cells with *N*-acetyl cysteine (NAC), a potent scavenger of H_2_O_2_,·OH, and HOCl and a precursor for the biosynthesis of glutathione [32-37], prior to the addition of cadmium or arsenite. At physiological pH, NAC abolished the ability of both cadmium and arsenite to trigger the phoshorylation of D-JNK, D-p38 MAPK, and D-ERK (Fig. 3, compare lanes 2–7 to lanes 11–16), but had no effect on the activation of D-JNK by sorbitol, an hyperosmotic stressor (Fig. 3, compare lanes 8 and 9 to lanes 17 and 18). Similar results were obtained using 6-Hydroxy-2,5,7,8-tetramethylchroman-2-carboxylic acid (Trolox^®^), a water-soluble derivative of vitamin E with antioxidant properties (not shown). We concluded, therefore, that as in mammalian cells [28], cadmium and arsenite activate *Drosophila *MAP kinases via ROS-dependent oxidative stress. These findings underscore the usefulness of the fly model to investigate cellular responses to environmental pollutants of relevance to human cells.

### D-MKK7 mediates the activation of D-JNK by cadmium and arsenite

**Figure 4:**
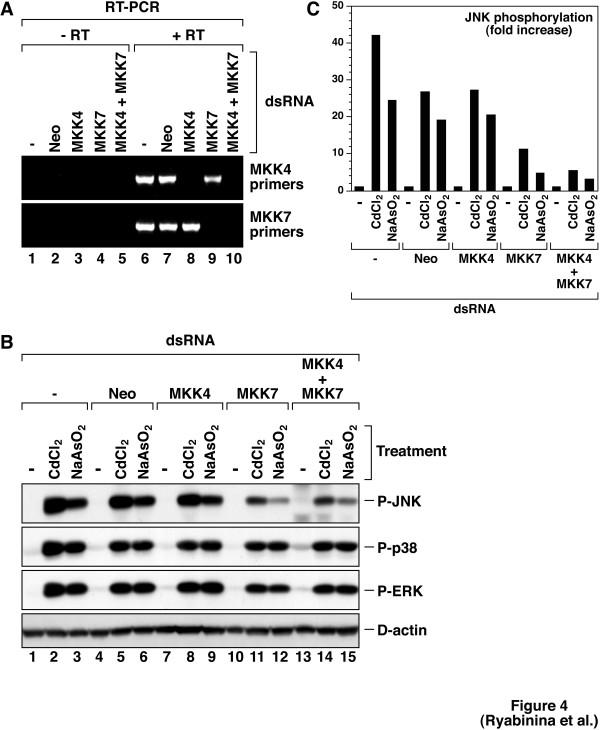
*Hep*/D-MKK7 mediates the activation of JNK by cadmium and arsenite. S2 cells were incubated, as indicated, in the absence ("-") of dsRNA, or in the presence of 100 μg per well of dsRNA specific to neomycin resistance gene ("Neo"), or 50 μg per well of dsRNAs specific to D-MKK4, or D-MKK7, or a mixture of both (50 μg each). Four days later, cells were treated with 200 μM CdCl_2 _or 200 μM NaAsO_2 _for 2 hours. Expression of D-MKK4 and D-MKK7 mRNAs was detected by RT-PCR using specific primers as indicated (A). "-RT", reactions without Superscript reverse transcriptase; "+RT", reactions with Superscript reverse transcriptase. Cell lysates were immunoprobed with antibodies recognizing P-JNK, P-p38, and P-ERK (B). An experiment representative of 3 repetitions is shown. A quantification of P-JNK (normalized for D-actin) is shown in (C). In this case, the basal levels of P-JNK in each RNAi group (lanes 1, 4, 7, 10, and 13 of Fig. 4B) are represented as "1".

To elucidate the respective contributions of D-MKK4 and D-MKK7 in the activation of D-JNK by cadmium and arsenite, we employed the method of RNA interference [30,31] to "knock down" the levels of D-MKK4 and D-MKK7 mRNAs either singly or in combination. Figure 4A demonstrates that dsRNA directed against D-MKK4 and D-MKK7 mRNAs (but not "control" dsRNA directed against a neomycin-resistance gene not present in S2 cells) reduced the levels of D-MKK4 and D-MKK7 mRNAs below detection by RT-PCR. Under these conditions, the D-MKK7 dsRNA, but not the D-MKK4 dsRNA, significantly reduced the respective abilities of cadmium and arsenite to activate D-JNK (Fig. 4B, compare lanes 2, 5, 8, 11, and 14 for cadmium and lanes 3, 6, 9, 12, and 15 for arsenite; Fig. 4C shows a quantification of the phosphorylation of D-JNK normalized for the levels in each lane of D-actin). Knocking down both D-MKK4 and D-MKK7 mRNAs in combination did not result in further inhibition of the activation of D-JNK by cadmium and arsenite (Fig. 4B, lanes 10–15). We concluded, therefore, that MKK7 is a major mediator of the activation of D-JNK by cadmium and arsenite.

### D-MEKK1 mediates the activation of D-JNK by cadmium and arsenite

**Figure 5:**
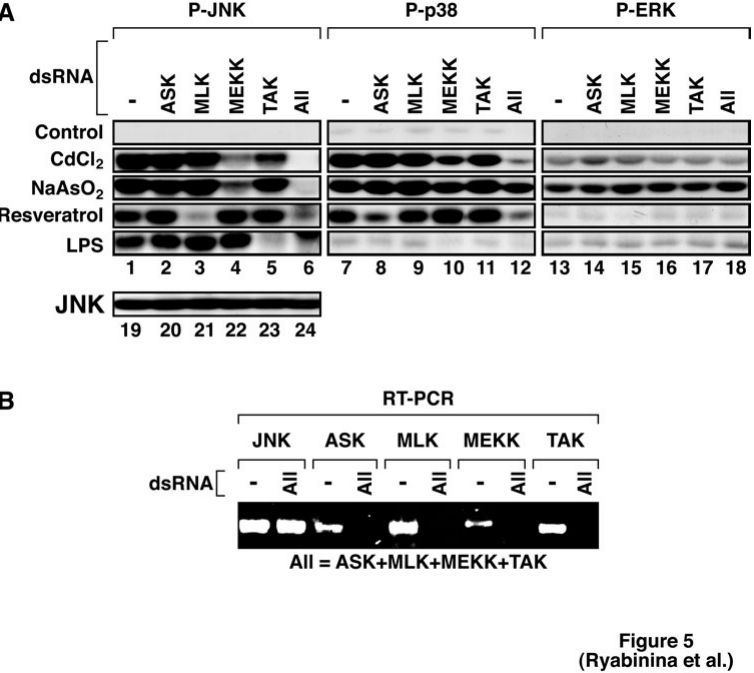
D-MEKK1 mediates the activation of JNK by cadmium and arsenite. S2 cells were incubated, as indicated, in the absence ("-") or presence of 15 μg per well of dsRNA specific to either D-ASK, D-MLK, D-MEKK1, or D-TAK or a mixture of all of them ("ALL") for 4 days followed by treatments, as indicated, with either 200 μM CdCl_2 _or 200 μM NaAsO_2 _for 2 hours, 200 mM resveratrol for 1 h, or 50 mM LPS for 10 min. Cells were harvested and analyzed for MAP kinase phosphorylation in immunoblot analyses using phosphoepitope-specific antibodies as indicated. Immunoblotblot analysis using an antibody recognizing JNK indicates equal protein loading (A). An experiment representative of 3 repetitions is shown. The levels of respective JNKKK mRNAs under conditios of RNAi using a mixture of dsRNAs specific for all four JNKKKs were detected in RT-PCR analyses and presented in (B). RT-PCR using primers specific for D-JNK demonstrates the lack of non-specific interference with D-JNK mRNA.

To elucidate the roles of various JNKKK members conserved between flies and mammals, we knocked down four JNKKK (D-ASK, D-MLK, D-MEKK1, and D-TAK), either singly or together (see Fig. 5B for an RT-PCR detection of the respective JNKKK mRNAs under conditions of RNAi). D-DLK was not included in the this analysis, as S2 cells have been found to be deficient in D-DLK expression [25]. Using appropriate stimuli, we were able to demonstrate efficient desired interference with the function of each of the chosen JNKKK. Specifically, (*i*) D-TAK dsRNA blocked the activation of D-JNK by LPS (Fig. 5, lane 5), a result consistent with the findings of Chen et al. [25]; (*ii*) D-MLK dsRNA blocked the activation of D-JNK by resveratrol and ethanol (Fig. 5, lane 3 for resveratrol and not shown for ethanol, *manuscript in preparation*); (*iii*) D-ASK dsRNA blocked the activation of D-p38 MAP kinase by resveratrol (Fig. 5, lane 8). Importantly, D-MEKK1 dsRNA reduced dramatically the activation of D-JNK by both cadmium and arsenite, while not affecting the activation of D-JNK by either resveratrol or LPS (Fig. 5, lane 4). (Although the experiment presented in Fig. 5 appears to demonstrate a role for D-TAK in the activation of D-JNK by cadmium, this result was not consistently reproducible.) Knocking down all four JNKKK together reduced the activation of D-JNK by cadmium and arsenite more efficiently than did knocking down of D-MEKK1 alone (Fig. 5, compare lanes 4 and 6), suggesting that JNKKK other than D-MEKK1 may contribute in a minor fashion to the full activation of D-JNK by toxic metals. Knocking down all four JNKKK proteins appeared to reduce the activation of D-p38 MAPK by cadmium significantly better than knocking down any single JNKKK (Fig. 5, lanes 7–12). Interestingly, knocking down all four JNKKK proteins did not reduce substantially the ability of arsenite to activate D-p38 MAPK (Fig. 5, lanes 7–12). Furthermore, knocking down JNKKK either singly or together did not affect the ability of cadmium or arsenite to activate D-ERK (Fig. 5, lanes 13–18).

### Lack of apparent involvement of Rho, Ral, Rac, cdc42, and Ras GTPases in the activation of D-JNK by cadmium and arsenite

**Figure 6:**
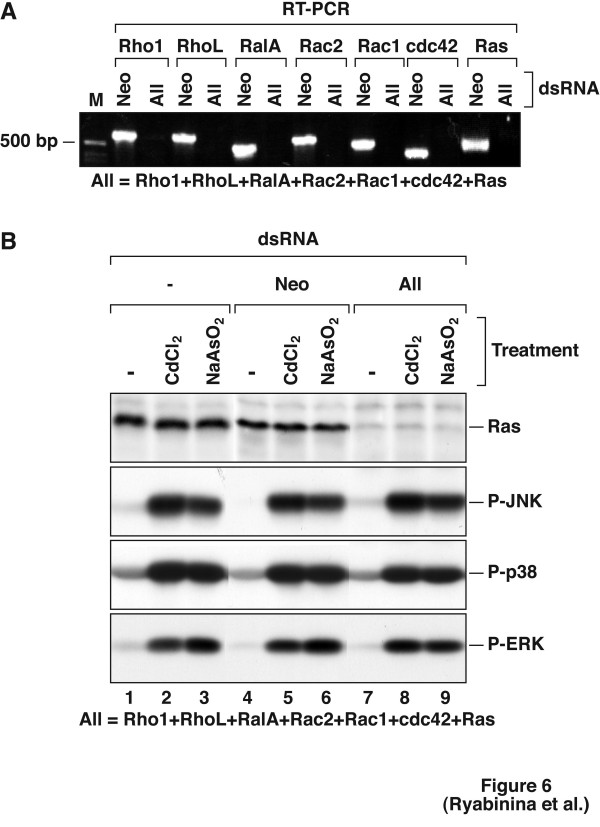
Lack of apparent involvement of Rho, Ral, Rac, cdc42, and Ras GTPases in the activation of D-JNK by cadmium and arsenite. S2 cells were incubated in the absence ("-") of dsRNA, or in the presence of 700 μg per well of dsRNA specific to neomycin resistance gene ("Neo"), or 100 μg per well of each of dsRNAs specific to small GTP-binding proteins from Drosophila (Rho1, RhoL, RalA, Rac1, Rac2, cdc42, and Ras) ("All"). Four days later, cells were treated with 200 μM CdCl_2 _or 200 μM NaAsO_2 _for 2 hours. Cell lysates were immunoprobed with antibodies recognizing P-JNK, P-p38, P-ERK, and Ras (B). Expression of Rho1, RhoL, RalA, Rac1, Rac2, cdc42, and Ras mRNAs was detected by RT-PCR using specific primers as indicated (A). An experiment representative of 3 repetitions is shown.

To address the potential involvement of small GTP-binding proteins in the regulation of D-MEKK1/D-MKK7/D-JNK signal transduction cascade stimulated by cadmium and arsenite, we used dsRNA directed against D-Rho1, D-RhoL, D-RalA, D-Rac1, D-Rac2, D-cdc42, and D-Ras mRNAs. Knocking down any of these mRNAs singly had no effect on the activation of D-JNK by either cadmium or arsenite (not shown). We therefore undertook to knock down all 7 GTPase mRNAs together. Figure 6A demonstrates that dsRNA directed against D-Rho1, D-RhoL, D-RalA, D-Rac1, D-Rac2, D-cdc42, and D-Ras mRNAs (but not neo^r ^dsRNA) reduced the levels of all these GTPase mRNAs below detection by RT-PCR. Furthermore, we could demonstrate a dramatic reduction of D-Ras protein levels in the cells treated with dsRNA directed against D-Rho1, D-RhoL, D-RalA, D-Rac1, D-Rac2, D-cdc42, and D-Ras mRNAs (but not neo^r ^dsRNA, Fig. 6B, lanes 7–9). The detection of D-Rho1, D-RhoL, D-RalA, D-Rac1, D-Rac2, and D-cdc42 proteins was hampered by the lack or poor quality of commercially available antibodies. Despite the efficient simultaneous silencing of the seven GTPase mRNAs (Fig. 6A), the respective abilities of cadmium or arsenite to activate all three MAP kinases investigated were not affected (Fig 6B, compare lanes 1–3 to 4–6 to 7–9). Thus, the experimental evidence available cannot demonstrtate a detectable involvement of D-Rho1, D-RhoL, D-RalA, D-Rac1, D-Rac2, D-cdc42, and D-Ras in the activation of D-JNK by cadmium or arsenite.

### Phylogeny analysis of the MEKK family

**Figure 7:**
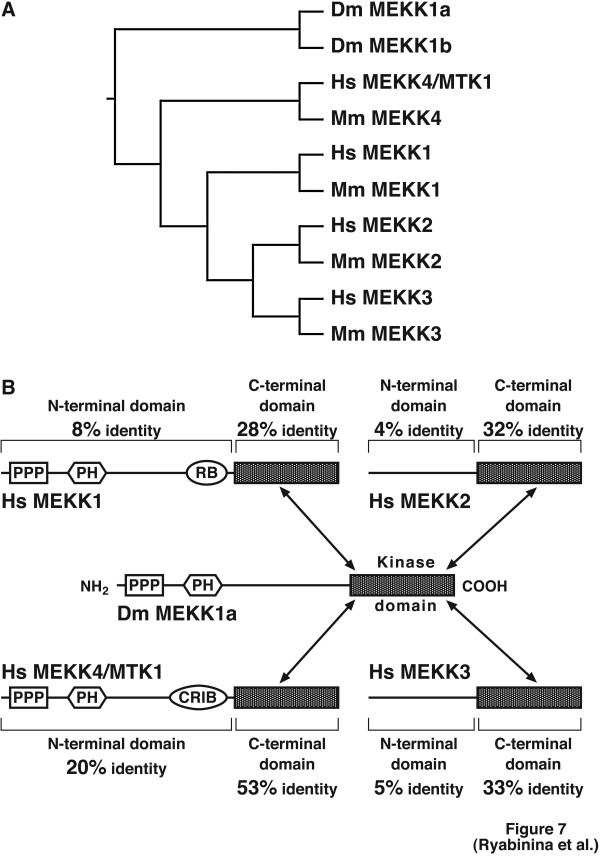
Comparison of the MEKK family: phylogenetic history and conserved domains. (A) Multiple sequence alignment of the MEKK family (including D-MEKK1a and D-MEKK1b, two alternatively-spliced variants of D-MEKK1 [38]) was done using Clustal X. Phylogeny was analyzed using Phylip and displayed using TreeView program. (B) Graphic representation of the selective divergence between the N-terminal regulatory domains and the C-terminal kinase domains of mammalian MEKKs from their Drosophila homologue D-MEKK1. Abbreviations (both A and B) are: Dm, *Drosophila melanogaster*, Hs, *Homo sapiens*, Mm, *Mus musculus*; PPP, proline-rich motif, PH, plekstrin homology motif, RB, Ras-binding motif, CRIB, cdc42/Rac-interactive binding motif.

To elucidate the closest homologies of D-MEKK1 in humans, we undertook a phylogeny analysis of *Drosophila*, human, and mouse MEKKs. Our analysis identified human MEKK4/MTK1 and mouse MEKK4 to be the closest homologues/orthologues of D-MEKK1, while the respective human and mouse MEKK2 and MEKK3 appeared to be the furthest in divergence (Fig. 7A). In addition to the high amino acid identity within the kinase domains of D-MEKK1 and MEKK4/MTK1 (53% identity, Fig. 7B), the sequence analysis revealed a substantial degree of conservation within the N-terminal regulatory domains of D-MEKK1 and MEKK4/MTK1 (20% identity, Fig. 7B). On the other hand, MEKK2 and MEKK3 appear to be furthest in divergence from D-MEKK1. It is also interesting to note that the MEKKs display distinct rates of divergence and selective conservation between their N-terminal regulatory domains. For instance, the N-terminal domain of D-MEKK1 displays two identifiable protein motifs that are present only in MEKK4/MTK1 and MEKK1: the proline-rich motif and the pleckstrin homology domain (Fig. 7B, [18,38]). The small GTPase-binding motifs of mammalian MEKK1 and MEKK4/MTK1 (a Ras-binding domain in MEKK1 and a cdc42/Rac-interactive binding motif in MEKK4/MTK1), however, are not present in D-MEKK1 (Fig. 7B, [18,38]). This suggests that small GTPase binding may not have been a requirement for regulation of the ancestral MEKK by oxidative stressors such as toxic metals and their compounds. Such a hypothesis is in agreement with the apparent lack of involvement of D-Rho1, D-RhoL, D-RalA, D-Rac1, D-Rac2, D-cdc42, and D-Ras in the activation of D-JNK by cadmium and arsenite in S2 cells (Fig. 6). Future mutational studies on D-MEKK1 combined with analysis of selective conservation within the N- and C-terminal domains in its mammalian homologues would contribute to the better understanding of the evolution of the JNK pathway signaling.

## Discussion

Investigation of toxicant-responsive signaling pathways relevant to human health using mammalian systems is sometimes complicated by the complex and often redundant nature of these pathways. Establishment of simpler model systems (e.g. using organisms of lower complexity) has proven useful in studying signaling pathways as long as these pathways are evolutionary conserved. Toxic metals and their compounds (such as cadmium and arsenite) are among the most ancient xenobiotic stressors affecting all forms of life. Therefore, it is highly probable that the cellular systems that identify and respond to toxic metals are fairly conserved within Metazoa. The fruit fly offers three major advantages as a model system to study cellular stress. First, *Drosophila *possesses many of the stress-induced signal transduction pathways relevant to man [25,29], the JNK pathway being one obvious example. Second, *Drosophila *displays, as a rule, lesser genetic complexity and redundancy of the same signaling pathways. For instance, the mammalian JNK family (JNK1, 2, and 3) is represented in the fly by a single member, D-JNK. Third, transient gene silencing by RNA interference (RNAi) is technically simpler, more efficient, and more affordable in *Drosophila *cells [30,31].

With the above rationales in mind, we undertook to identify upstream regulators of JNK that mediate the activation of the kinase by cadmium and arsenite. The principal novel findings we report here are that: (*i*) cadmium and arsenite activate D-JNK by means of oxidative stress similar to their action in mammalian cells [28]; (ii) the action of both cadmium and arsenite on JNK requires the engagement of upstream kinases at the level of JNKK and JNKKK; and (iii) D-MEKK1 is the predominant JNKKK that appears to mediate D-JNK activation in response to both cadmium and arsenite. We will discuss below the possible implications of these findings to the mammalian responses to toxic metals.

### Evolutionary conservation of metal toxicity through oxidative stress

We have reported previously that pretreatment of Rat-1 fibroblasts with NAC completely abolished the activation of JNK by both cadmium and arsenite [28], suggesting that oxidative stress through depletion of cellular glutathione is the *modus operandi *for JNK activation by these agents in mammalian cells. The ability of NAC (Fig. 3) and other antioxidants (such as lipoic acid and Trolox^®^, not shown) to inhibit JNK activation in *Drosophila *S2 cells underscores the suitability of the *Drosophila *model system for heavy metal-related toxicological studies, especially in terms of signal transduction pathways regulated by metal toxicants.

### Requirement for upstream kinases (JNKK and JNKKK) for the activation of D-JNK by toxic metals

Over the last decade, two mechanisms have been postulated for the activation of JNK by arsenite. One mechanism suggests that active engagement, by arsenite, of upstream kinases such as the JNKK MKK4 [28] and members of the MEKK family of JNKKK [39] is a prerequisite for the activation of JNK by this agent. Yet Cavigelli *et al*. [40] have found evidence that kinases upstream of JNK are not activated by arsenite. The experimental data suggested rather that arsenite activated JNK by means of inactivating a JNK phosphatase, thus increasing the steady-state levels of phosphorylated JNK in arsenite-treated cells [40]. The findings we report here lend support to the former mechanism.

In studying the regulation of JNK by cadmium, Matsuoka *et al*. [41] used mouse embryonic stem cells deficient in either MKK4 or MKK7 and concluded that both JNKKs are required for the full activation of JNK by cadmium. However, Ding and Templeton [42] used overexpression of dominant negative alleles of either MKK4 or MKK7 in mesangial cells to conclude that MKK7, but not MKK4, was responsible for the JNK activation by cadmium in these cells. Our results clearly identify a role for *hep*/D-MKK7 in the activation of D-JNK by cadmium (Fig. 4). The results from the use of RNAi to knock down the mRNA for D-MKK4 are subject, however, to more than a single interpretation. On one hand, the results tend to indicate the lack of apparent involvement of D-MKK4 in the activation of D- JNK by cadmium and arsenite (Fig. 4B). However, although the levels of D-MKK4 mRNA were brought down to levels undetectable by 30 cycles of PCR (Fig. 4A), the unavailability of suitable antibodies for detection of D-MKK4 precluded us from accessing the efficiency of the RNAi at the level of D-MKK4 protein. Thus, if D-MKK4 protein has a long half-life, the remaining amounts of D-MKK4 protein (even in the absence of available D-MKK4 mRNA) may have been sufficient to trigger JNK activation in cadmium- or arsenite-treated S2 cells. Further experiments are needed to clarify this point.

The need to identify the JNKKK(s) mediating the activation of JNK by cadmium and arsenite (and other oxidative stressors in general) in the genetically-simpler *Drosophila *system arises from the abundance of contradictory reports in the literature on the regulation of mammalian JNKKK by oxidative stress. Members of the mammalian MEKK family have been implicated by various research groups to mediate the activation of JNK by one or another oxidative stressor. Using dominant-negative alleles of MEKKs, Porter et al. [39] concluded that MEKK2, 3, and 4 (but not MEKK1) mediate the activation of JNK by arsenite. In contrast, mouse embryonic stem cell-derived from cardiac myocytes deficient in MEKK1 displayed attenuated activation of JNK in response to H_2_O_2_, a *bona fide *oxidative stressor [43]. However, in a recent report Cross & Templeton [44] described that mammalian MEKK1 is subject to *inactivation *rather than activation by oxidative stressors such as H_2_O_2_, menadione, and N-ethylmaleimide. These researchers identified the mechanism of this inactivation, namely the reversible glutathionylation of Cys^1238 ^of MEKK1 in response to oxidative stress [44]. Yet other researchers reported that ASK1 is a mammalian JNKKK responsive to oxidative stress [45]. In this case, the oxidation-dependent dissociation of an inhibitor of ASK1 (thioredoxin) was found to lead to the activation of ASK1, and subsequently, of JNK [45]. In summary, it appears that the mammalian response to oxidative stress may involve two different mechanisms of JNK activation, one mediated by MEKK-family member(s), and another by ASK1. One drawback of the published reports is that a systematic parallel investigation of the roles of MEKKs and ASK1 in the same cell type and using the same agents as oxidative stressors has not been done. Our results identified D-MEKK1 as a major JNKKK mediating the activation of D-JNK by cadmium and arsenite (Fig. 5). Furthermore, we did not find evidence for an involvement of D-ASK in mediating the activation of D-JNK by cadmium and arsenite (Fig. 5). One possible implication of these results is that the MEKK-dependent regulation of JNK by oxidative stressors is evolutionarily more ancient, whereas the ASK-dependent regulation of JNK by oxidative stressors has emerged more recently in evolutionary history.

Based on our findings and on the available published literature, we would like to propose that the ancestral pathway of regulation of JNK by cadmium and arsenite was dependent on the regulation of a MEKK type of JNKKK (represented by D-MEKK1 in the fly and by the MEKK family of kinases in mammals). In higher organisms the regulation of JNK appears to require a more complex modulation. This increased complexity is manifested by the expansion of the ancestral MEKK gene into a family of four MEKKs in mammals. Mammalian MEKK1 appears to have also acquired a regulatory Cys^1238 ^residue that allows for the oxidation-triggered inactivation of this kinase [44]. Our sequence analysis of MEKKs revealed that this cysteine residue is not present in D-MEKK1 or MEKK2, 3, and 4 (not shown). The need for more complex regulation of the JNK activity by oxidative stress in mammals has resulted also in the recruitment of ASK1 as another JNKKK. The interplay of MEKK(s) and ASK1 in mammalian cells is likely to determine the physiological outcomes (i.e. apoptosis *vs*. survival) of the cellular response to oxidative stress (see also refs [44] and [46] for discussion on the topic).

### Experimental procedures

#### Cell Culture

*Drosophila *Schneider (S2) cells (Invitrogen) were cultured at room temperature in *Drosophila *serum-free medium (DSFM, Invitrogen) supplemented with 20 mM L-glutamine (Invitrogen) and antibiotic-antimycotic reagent (Invitrogen).

#### Chemicals

Cadmium chloride, sodium-m-arsenite, resveratrol, sorbitol, *N*-acetyl cysteine, and lipopolysacharide (LPS; derived from *E. coli *0111:B4) were from Sigma-Aldrich.

#### Antibodies

Phosphoepitope-specific antibodies against JNK, p38, and ERK were from Cell Signaling Technology. These antibodies recognize the phosphorylated forms of *Drosophila *MAP kinases due to the conservation of the phosphoepitopes in metazoans. The antibody against human JNK1 (FL) (Santa Cruz Biotechnology) was used to detect D-JNK. For optimal performance with *Drosophila *extracts, this antibody needs to be used on naive membranes before any other antibody hybridization. The antibody against Ras (amino acid residues 31–43) was from Calbiochem. The epitope recognized by this antibody is completely conserved among metazoans. *Drosophila *actin was detected using the JLA20 monoclonal antibody from the Developmental Studies Hybridoma Bank (University of Iowa).

#### Cell viability

Viability studies using CellTiter-Blue™ Reagent (Promega) were performed according to manufacturer's protocol.

#### RNA interference (RNAi) in S2 cells

RNAi was performed according to Dixon laboratory's protocol [31]. S2 cells (3 × 10^6^) were plated in 1 ml of DSFM in 6-well tissue culture plates. Double-stranded RNA (dsRNA) was added and, after 1 hour of incubation, 1 ml of Schneider's *Drosophila *medium supplemented with 10% heat inactivated fetal bovine serum and antibiotic-antimycotic was added. Typically, cells were used for experiments 72 to 96 hours after the addition of dsRNA.

#### RNA isolation and Reverse Transcriptase-Polymerase Chain Reaction (RT-PCR)

Total RNA was isolated from 1 × 10^7 ^S2 cells using TRIZOL^® ^Reagent (Invitrogen) and according to manufacturer's protocol. First-strand cDNA was synthesized from total RNA using Superscript First-strand Synthesis System (Invitrogen). The same primers were used for cDNA synthesis and for detecting mRNA levels by RT-PCR. The sequences of all primers (except for the primers for *neo*^R^, see below) were exactly as described by Chen et al. [25] and contained a 5' T7 RNA polymerase-binding site. As a negative control for specificity of RNAi, we designed primers for a neomycin-resistance gene (*neo*^R^, not present in S2 cells), also with 5' T7 RNA polymerase-binding sites (forward primer: 5'-TAATACGACTCACTATAGGGAGACCATTGAACAAGATGGATTGCACG-3', reverse primer: 5'-TAATACGACTCACTATAGGGAGAGATGTTTCGCTTGGTGGTCG-3'). pcDNA3 plasmid was used as a template for the *neo*^R ^gene amplification. The following PCR program was used: an initial denaturation at 95°C for 5 min followed by 30 cycles of amplification (95°C for 1 min, 55°C for 1 min, and 72°C for 2 min) and an additional 10 min at 72°C. PCR products were cloned using TA Cloning Kit (Invitrogen) and sequenced for confirmation.

#### Generation of dsRNA for RNAi

Clones with confirmed correct sequences were used as templates for PCR. PCR products (ranging from ~500 to ~700 bp) served as templates for MEGAscript T7 transcription kit (Ambion) to make single stranded RNA (ssRNA). SsRNA products were ethanol-precipitated and resuspended in RNase-free water. SsRNAs were annealed by heating to 65°C for 30 min and then slowly cooling to room temperature to produce dsRNA. DsRNA concentration was measured by absorbance at λ_260 _nm and dsRNA samples were visualized on 1% agarose gel. DsRNAs were stored at -20°C.

#### Preparation of cell lysates and immunoblot analyses

To avoid potential post-lysis modifications or degradation of proteins of interest, the cells were harvested by direct lysis in 2 × SDS-PAGE sample-loading buffer, followed by heat denaturation at 95°C for 5 min and ultrasonic shearing. The electrophoretic separation of proteins in SDS-PAGE and electrotransfer onto PVDF membrane (Millipore) were performed using standard procedures. Immunoprobing with specific antibodies and enhanced chemiluminescent detection (DuPont NEN Research Products) were performed following the instructions of the respective manufacturers. For immunoblot quantification, appropriately nonsaturated film exposures were selected and scanned, and the scanned images were imported into IP Lab Gel (Molecular Dynamics) software for quantification.

## Authors contributions

OR carried out the experimental work and participated in its design. ES performed the sequence alignments and phylogeny analyses. MI conceived of the study, participated in its design and coordination, and drafted the manuscript. All authors participated in discussing and interpreting the results. All authors read and approved the final manuscript.
